# Why Does *Rhinopithecus bieti* Prefer the Highest Elevation Range in Winter? A Test of the Sunshine Hypothesis

**DOI:** 10.1371/journal.pone.0024449

**Published:** 2011-09-07

**Authors:** Rui-Chang Quan, Guopeng Ren, Jocelyn E. Behm, Lin Wang, Yong Huang, Yongcheng Long, Jianguo Zhu

**Affiliations:** 1 CAS Key Laboratory of Tropical Forest Ecology, Xishuangbanna Tropical Botanical Garden (XTBG), Chinese Academy of Sciences (CAS), Mengla, Yunnan, China; 2 Ecology, Conservation, and Environment Center (ECEC), Kunming Institute of Zoology (KIZ), Chinese Academy of Sciences (CAS), Kunming, Yunnan, China; 3 Department of Zoology, University of Wisconsin-Madison, Madison, Wisconsin, United States of America; 4 China Program, The Nature Conservancy, Yunnan, China; University of Bern, Switzerland

## Abstract

Environmental factors that affect spatiotemporal distribution patterns of animals usually include resource availability, temperature, and the risk of predation. However, they do not explain the counterintuitive preference of high elevation range in winter by the black-and-white snub-nosed monkey (*Rhinopithecus bieti*). We asked whether variation of sunshine along with elevations is the key driving force. To test this hypothesis, we conducted field surveys to demonstrate that there was a statistically significant pattern of high elevation use during winter. We then asked whether this pattern can be explained by certain environmental factors, namely temperature, sunshine duration and solar radiation. Finally, we concluded with a possible ecological mechanism for this pattern. In this study, we employed GIS technology to quantify solar radiation and sunshine duration across the monkey's range. Our results showed that: 1) *R. bieti* used the high altitude range between 4100–4400 m in winter although the yearly home range spanned from 3500–4500 m; 2) both solar radiation and sunshine duration increased with elevation while temperature decreased with elevation; 3) within the winter range, the use of range was significantly correlated with solar radiation and sunshine duration; 4) monkeys moved to the areas with high solar radiation and duration following a snowfall, where the snow melts faster and food is exposed earlier. We concluded that sunshine was the main factor that influences selection of high elevation habitat for *R. bieti* in winter. Since some other endotherms in the area exhibit similar winter distributional patterns, we developed a sunshine hypothesis to explain this phenomenon. In addition, our work also represented a new method of integrating GIS models into traditional field ecology research to study spatiotemporal distribution pattern of wildlife. We suggest that further theoretical and empirical studies are necessary for better understanding of sunshine influence on wildlife range use.

## Introduction

Hypotheses proposed to explain factors influencing the seasonal distributional changes of endotherms in response to winter have generally included one or more of the following three ecological processes: resource availability (food limitation), temperature effects on physiological function, and the risk of predation [1]–[4]. Spatial (altitudinal and latitudinal) and temporal variation in food resources may favor seasonal migration by forcing individuals out of unproductive areas during winter to exploit relatively richer areas [5]–[8]. Changes in precipitation and temperature can lead to conditions exceeding the range in which an individual can survive thus causing movements to more favorable areas [9]. Predation risk can vary by season causing migration to areas with fewer predators [3]. These and other factors can also interact with each other. Identifying the main factors controlling winter distribution patterns is complex and critical for predicting how animal populations will respond to changing conditions. However, the relative importance of biotic and abiotic constraints on distribution limits has not been clearly determined for most species.

Here we presented an investigation into a counterintuitive winter distribution pattern exhibited by the black-and-white snub-nosed monkey (*Rhinopithecus bieti)* in the mountainous region of the southeastern edge of the Tibetan Plateau. This species uses the high elevation areas of its home range during the winter season [10], [11]. Previous works have demonstrated that environmental temperature and the monkey's food resources decrease with increasing elevation and also decrease in winter compared to other seasons (e.g. [12], [13], [14]). In addition, environmental temperature declines and snow cover increases with increasing elevation further increase the metabolic demands of the species in high elevation and low food areas [15]. Additionally, there is no evidence to suggest that this pattern is in response to avoiding predators. *R. bieti* is semi-terrestrial primate with a large body size (adult male with 30 kgs and adult female with 15 kgs) and large canine size (male of 22 mm and female of 15 mm above the teeth ridge), making it difficult for predators to subdue. Potential predators in the area include a buzzard (*Buteo spp*., a potential predator to infants [16]), black bear (*Selenarctors thibetanus*) and snow leopard (*Uncia uncia*) [17], Up till now, the only confirmed predator of the species is human beings. However, humans are not the cause of the monkey's winter distributional pattern because hunting monkeys is now illegal and human activities in the area such as caterpillar fungi collection, livestock grazing and movement of people between villages are consistent along the elevation gradient and largely cease in winter (e.g. [14]).

We propose a sunshine hypothesis to explain the winter distribution pattern of *R. bieti*. In areas of extreme topography such as our study site, solar radiation can vary dramatically, which might have a strong direct influence on local temperature and vegetation patterns, thus indirectly influencing the monkey's distribution. In general, both soil and air temperatures are significantly higher on south slopes versus north ones [18], [19] and solar radiation plays an important role in many physical and biological processes of animal [20].

We first set out to demonstrate that there is a statistically significant pattern of high elevation use by the monkeys during winter months. We then ask whether this pattern can be explained by certain environmental factors, namely temperature, sunshine duration and solar radiation. Next, we limit our focus to the winter range only and ask the same questions: is there a strong habitat preference within this range and can this preference be attributed to sunshine duration and solar radiation. Finally, we conclude with a possible ecological mechanism for this high elevation use pattern.

## Results

### Winter Range along Elevations

**Figure 1:**
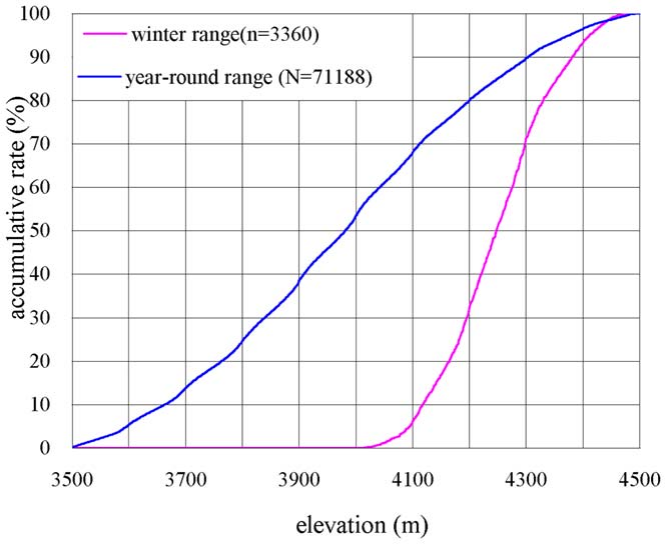
The cumulative percentage of area for winter home range and year-round home range along with elevation (3501–4500 m).

The proportion of the yearly home range area used was almost equal from 3500 to 4500 m, however, the elevation from 4100 to 4400 m formed almost 87% of the winter range, but it only accounted for 29% of the total yearly home range size and 30% of the elevational zones (Fig. 1). The area below 4100 m (3500–4100 m), which represented 68% of the total range size and 60% of the elevational zones, was almost completely avoided by the monkeys in winter. The monkeys prefer the higher elevation zones within year-round home range in winter (χ^2^ = 5938.92, df = 4, p<0.001).

### Environmental Factors across Home Range

#### Sunshine duration and radiation along elevation

**Figure 2:**
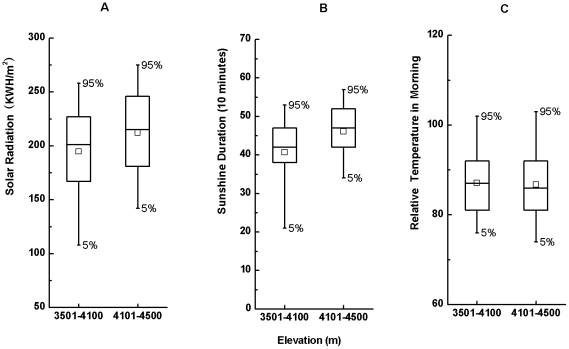
Mean radiation (A) and duration (B) of sunlight, and relative temperature (C) across the winter range (4101–4500 m) and non-winter range (3500–4100 m). Horizontal lines represent medians and quartiles; boxes represent means.

During our observation period (1^st^ Nov 2006 to 10^th^ Feb 2007), solar radiation was positively related to elevation (R^2^ = 0.05, t = 66.42, p<0.001). The mean solar radiation (212±42.6 KWH/m^2^, n = 24642; from1^st^ Nov 2006 to 10^th^ Feb 2007) in the monkeys' main winter range (4101–4500 m) was significantly higher than non-winter range (194±44.4 KWH/m^2^, n = 52486; 3501–4100 m) (Fig. 2a, t = −52.09, p<0.001).

Sunshine duration was also positively related to elevation (R^2^ = 0.12, t = 103.04, p<0.001). The mean sunshine duration (460±78 mins, n = 24642) in the monkey's main winter range (4101–4400 m) was significantly higher than that of the non-winter range (400±99 mins, n = 52486; 3500–4100 m) (Fig. 2b, t = −75.34, p<0.001).

#### Surface temperature

In general, temperature decreased with elevation increasing (R^2^ = 0.01, t = −27.71, p<0.001). However the temperature at our study site did not decrease uniformly with elevation increasing, and it reached lowest between 3901 and 4100 m, increased again at higher elevations. The mean temperature (relative temperature: 86.7±9.9, n = 24642) in the monkeys' main winter range (4101–4400 m) was almost the same as that of the non-winter range (87.1±8.9, n = 52486; 3501–4100 m) (Fig. 2c).

### Habitat Preferences within the Winter Range

During the 2006–07 winter, the winter range of the monkeys mainly spanned from 4100 to 4400 m, and the total forest area used was 2.10 km^2^, which spread over nine MAPs (Appendix S1, Table 1). Even so, the species was still not uniformly distributed across this limited winter range (χ^2^ = 53.9, df = 8, p<0.001). Three of the MAPs (No. 2, 8, 9) were used most intensively and 6 were used less intensively (Table 1).

**Table 1:** Description of 9 MAPs identified as winter range for monkeys during winter of 2006–07*.

MN	RU	Area (km^2^)	ME (m)	MSD (min.)	MSR (KWH/m^2^)	MRT
1	1	0.24	4298.2 (±78.9)	401 (±37.9)	159 (±20.8)	76 (±1.8)
2	17	0.24	4228.1 (±73.2)	470 (±28.8)	241 (±18.4)	85 (±2.6)
3	3	0.23	4140.9 (±69.1)	328 (±79.8)	152 (±24.0)	78 (±1.5)
4	1	0.29	4290.1 (±90.1)	370 (±92.0)	151 (±24.9)	75 (±2.0)
5	2	0.11	4251.1 (±60.1)	374 (±64.6)	180 (±27.1)	84 (±3.0)
6	3	0.27	4356.4 (±63.3)	437 (±39.6)	203 (±31.0)	80 (±5.2)
7	4	0.30	4267.8 (±50.9)	433 (±32.2)	188 (±21.2)	79 (±2.4)
8	9	0.13	4171.2 (±40.6)	488 (±52.2)	228 (±19.3)	83 (±1.8)
9	17	0.30	4179.7 (±65.2)	503 (±20.6)	235 (±14.8)	87 (±2.4)

*MN: MAP no.; RU: range use (days); ME: mean elevation (±SD), MSD: mean sunshine duration (±SD) on Dec 22^nd^, 2006; MSR: mean solar radiation (±SD) from Nov 1^st^, 2006 to Feb 10^th^, 2007; MRT: mean relative temperature (±SD).

### Environmental Factors within Winter Range

#### Sunshine duration and radiation

**Figure 3:**
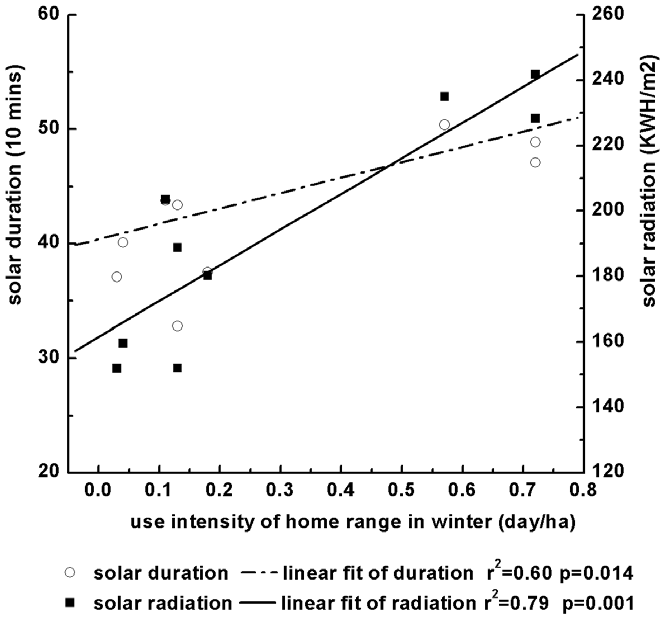
Relationship between range use intensity (days/ha) and sunshine duration and radiation within the winter range (mainly 4100–4400 m).

Winter habitat use intensity within the 9 MAPs was positively correlated with solar radiation (R^2^ = 0.79, t = 5.17, p = 0.001) and sunshine duration (R^2^ = 0.60, t = 3.25, p = 0.014) (Fig. 3).

The MAPs with average solar radiation more than 220 KWH/m^2^ were preferred (χ^2^ = 51.2, df = 1, p<0.001). The total area of these three MAPs was only 0.66 km^2^ (31.4% of total wintering range), which accounted for 75% (43 out of 57 days) of the time the species spent in their winter range. The time that the species spent in the remnant 1.44 km^2^ (68.6% of total wintering range), which has an average solar radiation less than 200 KWH/m^2^, only accounted for 25% of the total time in winter range.

The largest difference of the average duration of sunlight among the nine MAPs was nearly 170 min (minimum of 328 min and maximum of 503 min), and the monkeys preferred the area with a longer duration of sunlight.

#### Tree DBH and Height

There was no significant difference between use intensity of winter range and range quality measured as tree DBH (F_3, 229 = _1.81, p = 0.146), even though the tree height was higher in low use intensity areas (F_3, 228 = _36.85, p<0.001).

#### Snowfall

**Figure 4:**
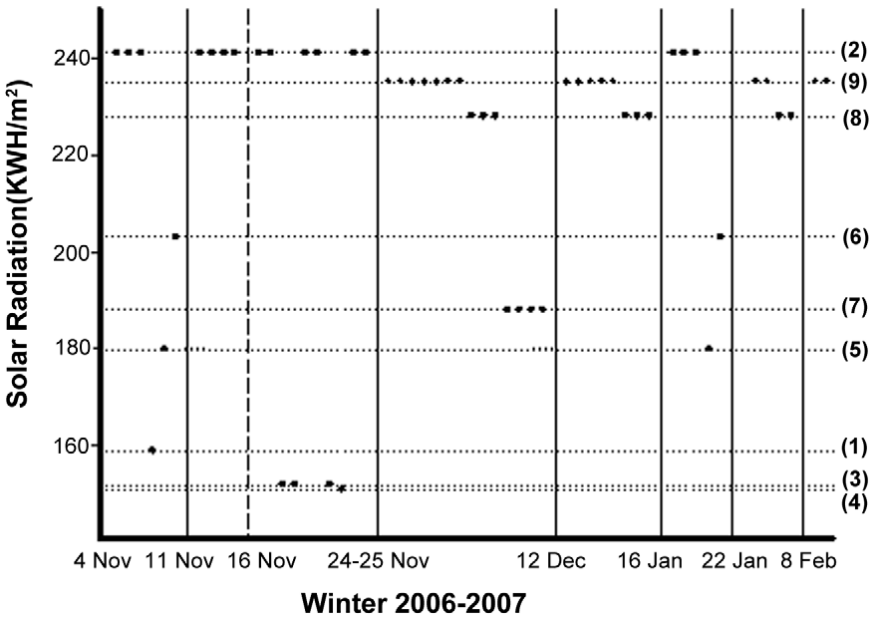
Monkeys were found in MAPs with highest solar radiation following snowfalls during the snowy period of our winter 2006–2007 survey. MAPs are arranged according to mean solar radiation and are indicated by number in parenthesis on the right vertical axis (Appendix S1, Table 1). Black dots indicate the locations of monkeys. Snowfalls are indicated by vertical lines and correspond to dates on the x-axis. The dotted vertical line indicates a short, 1–2 h snowfall during the day.

**Figure 5:**
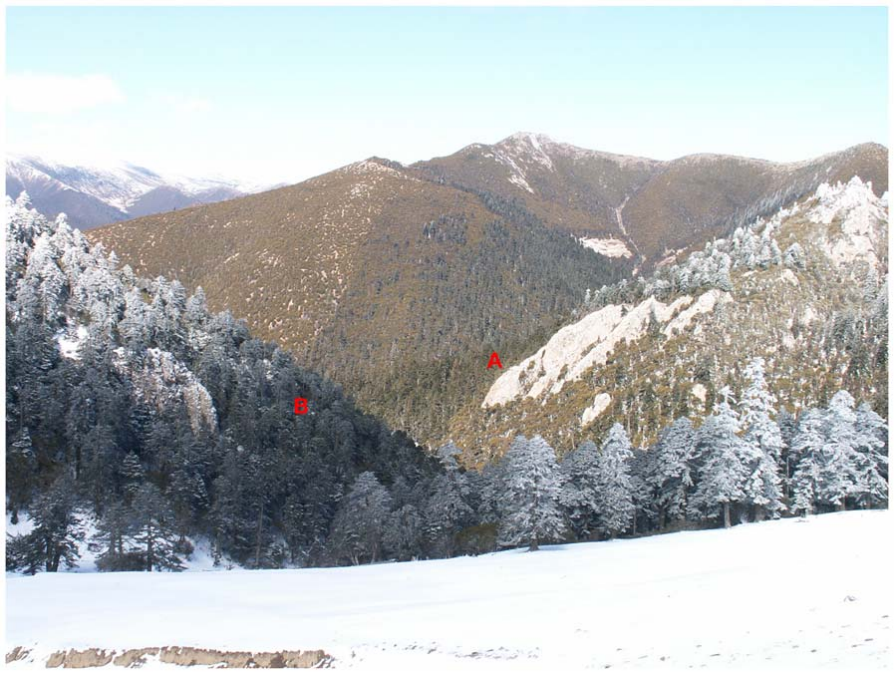
Two neighboring ranges (A and B) with different amounts of solar radiation on the second day after snow. A: there was almost no snow covering the tree canopy in a range with an average solar radiation about 241 KWH/m^2^ (MAP 2, see Table 1); this is one of the preferred habitats immediately after snow (see Fig. 3). B: a snow layer of about 5–7 cm covered most of the tree canopy in a range with solar radiation of 180 KWH/m^2^ (MAP 5); the canopies were usually not available for foraging for about 3 to 4 days after snow.

During the winter, the monkeys spent about 51.2% of time feeding and 19.7% sunbathing. As snow accumulates on tree surfaces, snowfalls seem to have a strong impact on monkey behavior by restricting activities such as food searching and sunbathing. From November 4, 2006 to February 11, 2007, there were 8 times of snowfall. In the days following each of snowfalls (1–2 days), monkeys almost always used the MAPs with high solar radiation (χ^2^ = 15.3, df = 1, p<0.001), regardless of where the monkeys were observed before the snowfall (Fig. 4). Snow accumulation on tree canopies differed between areas differing in solar radiation as quickly as the second day following a snowfall (Fig. 5). Areas with high solar radiation only accumulated a small snow layer on tree surfaces compared to low solar radiation areas which retained snow covered for several days following a snowfall. Therefore, it appeared that the monkey's MAP selection was correlated to the duration of snow cover on tree branches which is related to solar radiation.

## Discussion

To our knowledge, this is the first study to relate sunlight with winter space distribution of monkeys in temperate, high elevation areas. The yearly home range of the species was almost evenly distributed along elevations spanning from 3500–4500 m, but showed a strong preference for the high elevation in winter, represented by the nine MAPs with a mean elevation of 4200 m. Within the monkey's entire home range, solar radiation and sunshine duration were correlated with increasing elevation, and were greater in the winter range compared to the non-winter range. Temperature significantly decreased with increasing elevation, however the mean temperature was the same between the winter and non-winter ranges. In our study area, food availability declines with increasing elevation [14] and the duration of snow cover on the ground increases with elevation further, limiting food availability. Since there is almost no difference in temperature between winter and non-winter ranges, and food is also less abundant for the whole range (3500–4500 m) in winter. It seems counter-intuitive that the monkeys would expend the energy to go to higher elevations in the winter. Thus, the monkey's winter range appeared to be driven entirely or at least partially by solar radiation and sunshine duration.

The winter range of the species, represented by the nine MAPs, was distributed in almost the same high elevation zone (mainly 4100–4400 m), and the total area was 2.10 km^2^. However, the monkeys showed a strong preference for three of the MAPs (2, 8, and 9, see Table 1), which only account for 31.4% of the total winter area, but represented 75% of the time the species spent in winter. We found that monkeys preferred the MAPs that had higher solar radiation and sunshine duration. Although the MAPs have the same elevation span, the largest solar radiation difference between the most intensively (241 KWH/m^2^) and least (151 KWH/m^2^) used MAPs was about 90 KWH/m^2^, and the difference between the longest (503 mins) and shortest (328 mins) sunshine duration was also more than 170 min. The variation of solar radiation and sunshine duration at the same elevations may be attributed to a topographical difference, since southern slopes usually have more sunshine duration. Previous research suggested that tree DBH and height were possible factors to influence winter range use in terms of safety (e.g. [16], [21]), but we found no correlation between DBH and range use intensity and a negative correlation between tree height and range use intensity. These conflicting results may be because the areas with larger trees usually had a shorter sunshine duration, which may outweigh the benefit of large trees at our study site. We also found that snow cover lasted longer in tree canopies in MAPs with lower solar radiation and sunshine duration. We recorded monkeys moving from MAPs with lower solar radiation to MAPs with higher solar radiation immediately following snowfall events, presumably because the higher solar radiation melted the snow faster and created more locally abundant food. Therefore, it appears that within their winter range, the monkey's distribution pattern may be in response to food availability. However, if food availability was the sole driving factor explaining their distribution, they should be in lower elevations during winter where food abundance is higher [14], [21]. Since this is not the case, we believed that a factor in addition to food availability was responsible for this pattern.

For other species, changes in distribution in response to winter have been explained by avoiding unfavorable temperature [7], [22], [23], moving to areas of high food availability and escaping from predators/disturbance [1]–[4]. These factors alone do not explain the *R. bieti*'s winter distribution pattern. We concluded that the monkeys move in response to solar radiation and sunshine duration alone or in concert with other unknown factors. While these solar factors seem to be correlated with food abundance, we also believed that the monkeys might seek out the sun directly. On several occasions, we observed monkeys spending up to 20% their time sunbathing (Quan unpublished data). Exposure to the sun may improve their winter survival by minimizing the energetic cost of thermoregulation (e.g. [24], [25], [26]), especially for juveniles. Because small-sized juveniles have a high surface-to-volume ratio they are expected to derive greater benefit from exposure to sunlight by having a higher rate of solar heat gain relative to their body mass. Sunlight may also help to control skin diseases by over heating and killing or stunning some small ectoparasite, such as lice and mites [27], [28].

The winter distribution pattern exhibited by *R. bieti* seems unique among mammals, but not unique among species to this region. Data on winter range use in species from adjacent areas with similar variation in elevation and climate also show the same pattern of high elevation zone use in snow-covered winter [10], [11]. We also observed a widely distributed primate species, *Macaca mulatta*, use elevations even above 4500 min our study area in winter of 2005 and 2006–2007 (Quan *personal obs.*). In addition, other non-primate endotherms in the same and nearby regions such as takin (*Budorcas taxicolor*), wild yak (*Bos grunniens*) and Sichuan jay (*Perisoreus internigrans*) were also reported to use higher elevations in winter [29]–[32]. This pattern extends beyond the Trans-Himalayas to include short-toed tree-creepers (*Certhia brachydactyla*) from temperate forests in the southwestern Palaeartic [24] and sika deer (*Cervus nippon yesoensis*) from temperate Japan [6]. All these examples suggested that this might be a common phenomenon for some mammals and birds species in temperate montane regions. Future work should focus on the role of solar radiation in winter range distributions to determine whether a “sunshine hypothesis” explains the distribution patterns of some endotherms in temperate mountain areas.

It is possible, however, that sunshine is disproportionately important in our study area compared to other temperate montane regions. Because of the high elevation (>4000 m on average) and low latitude (approximately 27°–40°N), the Tibetan Plateau has an unusually high amount of sunshine compared to the small amount of sunshine in other temperate regions which usually have lower elevations with higher latitude or higher elevations with higher latitude. This difference is further magnified by high proportion of sunny days in our study area and longer day length in winter due to its low latitude. For example, at our study site (29°N), 80% of the days are cloud-free with 10–11 h of winter daylight compared to 35-40% cloud-free days and 9–10 h of daylight in southern Europe (40°N), and 70% cloud-free days and less than 5 h of daylight at latitudes higher than 50–55°N [24]. Future work in the Tibetan Plateau and adjacent areas in the mountains of southwest China biodiversity hotspot contrasted to other montane forested areas in regions like Japan and Spain [6], [24], [25] should help elucidate whether sunshine is important only to our study region or if it's more globally important.

Finally, future work should experimentally measure the direct benefits that the animals gain from sunshine, such as altitude-related differences in solar energy gained by pelage. Black fur is thought to have a greater capacity to gain solar heat [33], [34]. Many animals in our study region such as *R. bieti*, yak, giant panda, and takin have dark fur and could be useful subjects for future experiment research.

### Conservation implications

*R. bieti* is an important flagship species of the “Mountains of Southwest China Biodiversity Hotspot”. It was categorized as Endangered (C1) on the IUCN Red List of 2010 [35], and is in the First Class of State Key Protected Species in China. Previous surveys have shown that deforestation and hunting are major threats to the survival of *R. bieti* throughout its range [36], [37]. However, our study indicates that climate change might also be a potential threat to the monkey's survival for the following reasons: 1) the species has an extremely high winter range (>4100 m), and high elevation environments are highly susceptible to global climate change [38], [39]; 2) due to the high frequency of snowfalls in winter, the monkeys are heavily reliant on clear cloudless days following a snowfall for sunshine to heat trees and melt snow for food availability; 3) the monkeys may also rely on solar energy to behaviorally buffer climate and energy stress in cold winter. If the frequency of cloudless days decreases, it will be a disaster not only to the monkeys, but also to some other mammals and birds species wintering in Tibet Plateau and the surrounding high mountain regions.

### Conclusion

We have empirically demonstrated that variation in sunshine along an elevation gradient was significantly correlated to the spatial distribution pattern of *R. bieti* in winter in a mountainous region. These results are in contrast to previously invoked hypotheses to explain seasonal migration patterns in other species such as food abundance, temperature, and predators. Our data analysis using a new GIS solar radiation model, allowed us to formulate a “sunshine hypothesis” to explain *R. beiti's* counterintuitive high elevation range selection in winter. Our “sunshine hypothesis” and model can also be applied to other wildlife in this and other mountainous regions, and highlight the need for both theoretical and empirical studies to better understand the influence of sunshine on wildlife range distribution in the era of global climate change.

## Methods

### Species and Study Area

The black-and-white snub-nosed monkey (*Rhinopithecus bieti*) is a “flagship” species endemic to the Trans-Himalayas between Yunnan and Tibet, bounded by the upper Yangtze and Mekong Rivers. It was categorized as Endangered (C1) by the 2010 IUCN red list [35] with a total population less than 1700 individuals [36], [37]. We carried out our study at the southeastern Tibetan Plateau (Appendix S1, 98°34′–40′E, 29°13′-18′N) with an elevation range between 3200 m and 4500 m. The dominant vegetation types in the area are coniferous forests, evergreen broad-leaf forests, and some deciduous broad-leaf forests and shrubs [17]. Fir trees (*Abies spp.*) are the most dominant tree species spanning the entire range, and evergreen broad-leaf trees and deciduous broad-leaf trees mixed with fir trees are mainly distributed in lower elevations (<3900 m). Understory vegetation is scarce especially above 4000 m and dominated by rhododendron (*Rhododendron spp.*). The Chinese national highway near the winter range of the species, permanent local settlements with some reaching up to 4000 m, trails between settlements, and local people's activities such as logging, yak and sheep grazing, caterpillar fungus [*Cordyceps sinensis*] and firewood collection in the forest, represent most of the potential anthropogenic disturbance factors to affect the monkey's range distribution.

Regardless of the topographic and weather factors, the sunshine duration at 29°15′N, the central belt of the study site, decreased from 648 minutes in Nov. 1^st^ to 606 minutes in Dec. 22^nd^, 2006, and then increased to 650 minutes in Feb. 10^th^, 2007. 80% of the winter days are cold and cloudless with daily average temperature <−4°C.

### Delineating Range Use

#### Total home range

To estimate the monkey's total home range in our study site, we partitioned the study site into several small watersheds, which were bounded by mountain ridges, using ArcGIS software v. 9.2 (ESRI, Redlands, CA, 2006) with the aid of a digital elevation model (DEM). Each watershed was divided into two hill-slopes by the stream. The year-round total home range was defined by the hill-slopes where monkeys had previously been recorded from our field surveys during 2006–2008 (8 months over 3 years) and previous studies (e.g. [14], [37]). Areas lower than 3500 m or higher than 4500 m, and non-forest patches larger than 1 km^2^ were excluded from these hill-slopes (Appendix S1).

#### Winter home range

We conducted surveys for four months during the winter (October 7, 2006 - February 11, 2007), by mainly walking along two selected survey routes at 3500 m to 4500 m almost every day to look for the monkey groups. We determined the winter range of the monkeys by direct observation and by traces left by the monkeys, such as footprints, excrement, and broken branches and twigs on the snow-covered ground. Monkeys are scared of humans. To avoid influencing their range distribution, we sketched their approximate activity area each day on a 1∶100,000 map from a distance of about 200–500 m away from the monkey group. Once the monkey group left the area, we used a hand-held GPS to outline the activity area by walking along the edge recorded on the map. At the end of the season, points were linked together to form minimum active polygons (MAP), and all the MAPs together represented the total winter home range. We recorded the number of days the monkey group, consisting of about 200 individuals split into around 20 family units, stayed in each MAP either continuously or intermittently. If the monkey group stayed in an area less than one day and no night sleeping site was found, this was treated as a traveling route between ranges (MAPs). From October 2006 to February 2007, a total of 57 days distributed in 9 MAPs were observed.

The field surveys were permitted by Forestry Department of Tibet Autonomous Region (permit number: [2006]-3) and the Management Bureau of Hongla Snow Mountain National Nature Reserve All procedures during field surveying were in accordance with the requirements of “Law of the People's Republic of China on the Protection of Wildlife”.

### Environmental Factors across the Home Range

#### Temperature

We wanted to determine the pattern of temperature variability along the monkeys' entire home range; however, there are not enough weather stations to measure the temperature at a fine enough resolution within our study area (3500–4500 m). Therefore we used the thermal infrared sensor data (wavelength of 10.40–12.50 µm) acquired on 25 December 2006 from Landsat 5 to represent relative surface temperature in mid-morning over the study area, as this method has proven feasible by previous studies (e.g. [40], [41], [42]). We refer to this as relative temperature since the thermal image has no units and simply represents the relative differences in temperature among locations.

#### Solar radiation

The insolation of a certain position is determined by the weather, solar azimuth, solar altitude, and the neighborhood topography. We simulated the solar illumination of a surface at a certain cloud-free time (Environmental Systems Research Institute, ESRI) by inputting the solar azimuth and altitude into the Hillshade function in ArcGIS and creating a hillshade raster from a digital elevation model (DEM). The solar azimuth (*a*) and altitude (*h*), which are determined by date, time, and geographic latitude (*φ*) of a surface, can be calculated by Eqs. (1–3).
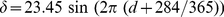
(1)

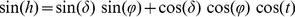
(2)

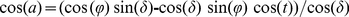
(3)where: *δ* is the solar declination, which varies with Julian day *d*; *t* is the solar hour angle at a certain time on day d, specially, at the solar noon, *t* = 0; *a* and h refer to the solar azimuth and altitude with the solar hour angle of t and latitude of ϕ.

At sunrise and sunset, the solar elevation is equal to zero. Eq. (4–5) is derived from Eq. (2) to calculate the hour angle of sunrise (*t_sr_*) and sunset (*t_ss_*).
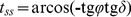
(4)

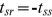
(5)


To approximate the duration of direct sunlight on a certain day, a discrete time series *t_i_* (*i* = *−k*,..., −1, 0, 1, ..., *k*; 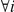, *t_sr_*≤*t_i_*≤*t_ss_*), which is defined by Eq. (6), was used to represent this day from sunrise to sunset.
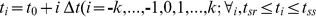
(6)


In Eq. (6), *t*_0_, equal to zero, is the hour angle at solar noon; *Δt* is a given interval of time measured in hour angle (1° = 4 minutes); k is an integer, which is less than the quotient of *t_ss_* divided by *Δt*.

Using the Hillshade function, a series of hillshade rasters can be created to simulate the solar illumination of the study area at time *t_i_* (*t_sr_*≤*t_i_*≤*t_ss_*). Thus the duration of sunlight (unit: minute) could be approximated through these hillshade rasters. If the value of a cell in a hillshade raster is greater than zero, it means that this cell is in sunlight at that time. Suppose there are n (0≤n≤2k+1) hillshade models valuing greater than zero for a certain cell, the duration of sunlight of this cell is approximately 4nΔt minutes.

In this study, we approximated the duration of direct sunlight of the whole study area on the winter solstice on December 22, 2006, because the duration of direct sunlight on this day is the shortest within a year, which represents the extreme minimum in sunlight duration for wildlife (animals and vegetation). A DEM at 25 m resolution was derived from the 1∶100,000 scale topographic map. Three parameters were given below (Eq. (7–9).
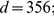
(7)

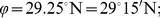
(8)

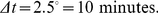
(9)


**Figure 6:**
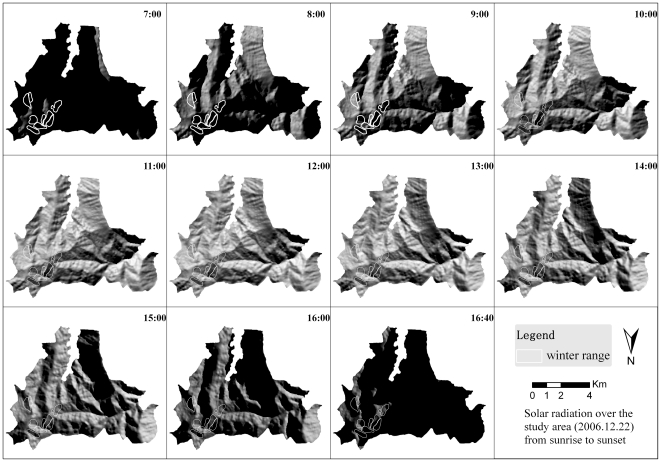
The pattern of solar illumination throughout the monkey's entire home range (3500 to 4500 m) from sunrise (7:00 am) to nearly sunset (16:40 pm) on December 22, 2006. Areas with dark/light color indicate where there is no sun/sun exposure respectively. Higher elevation areas usually have a longer duration of sunlight (the sunlight both begins earlier and ends later in higher areas), indicated by light color.

Fig. 6 shows the simulation of solar illumination from sunrise to sunset on December 22^nd^, 2006 over the entire study area.

We calculated the amount of solar radiation in KWH/m^2^ from Nov 1^st^, 2006 to Feb 10^th^, 2007 over the total home range using the function Area Solar Radiation in ArcGIS (latitude: 29.25°N; Sky size: 200; start day: Nov 1^st^, 2006; end day: Feb 10^th^, 2007; Day interval: 1day; Hour interval: 0.25 hour).

### Environmental Factors across the Winter Range

#### Solar radiation and duration

We used the same methods outlined above to calculate the solar radiation and duration for the MAPs on the dates when we observed monkeys during the winter 2006–07 field season. For each MAP we calculated the average sunshine duration and intensity across all dates when monkeys were present and used these values for statistical analyses. Appendix S2 shows the simulation of solar radiation and sunshine duration of the monkey's winter ranges (represented by 9 MAPs).

#### Tree DBH and height

Previous work showed that environmental temperature, disturbance, and vegetation were not factors to determine the monkey's winter range distribution [43], [44], other ecological needs such as sleeping sites and sleeping trees (DBH [diameter at breast height] and height) are rather important [16], [21]. Therefore, we did not survey folivorous food resources since it has not been shown to be a causal factor in range use and has been shown to decline with increasing elevation and latitude within the distribution range [12]–[14]. We surveyed tree DBH and tree height within 20×10 m quadrats in the winter range in areas that represented different intensities of range use (>10 d, 6–10 d, 3–5 d and <3 d, 6 quadrats for each level).

#### Snowfall

Snow cover on trees may affect the range distribution of *R. bieti* by restricting food availability. We surveyed and compared the snow cover status on tree canopies in different winter range MAPs by taking photos at the same time of the day on subsequent days following a snowfall. This proved to be the best method for assessing snow cover on canopies as opposed to directly measuring the percentage of snow cover in the tree-crowns given that canopy height generally exceeds 15 m.

### Statistical Analysis

#### Assessing range use patterns

To analyze range use along elevation, we divided the home range into five 200 m elevation intervals (3501 to 4500 m) and calculated the total numbers of days the monkeys were observed in each elevation interval (about 8 months for the yearly home range over 2006–2008, and more than 3 months for the winter home range over winter 2006–2007). Then, we used a chi-square test to determine if the monkeys prefer the higher elevation zones in winter [45].

#### Correlation analysis of environmental factors and elevation across total range

We calculated Pearson correlation coefficients to test whether temperature, solar radiation and sunshine duration are correlated to elevation. We then calculated the mean values of these three variables for the winter (4101–4500 m) and non-winter ranges (3501–4100 m) and used t-tests to check for differences [45].

#### Correlation analysis of environmental factors and habitat use within winter range

For each of the 9 MAPs, we calculated range use intensity by dividing the number of days it was used by the total area. We then used correlation to test whether habitat use intensity was related to either sunshine duration or solar radiation. We used ANOVA to answer if the tree DBH and height were associated with range use intensity categories (>10 d, 6–10 d, 3–5 d and <3 d).

To demonstrate the influence of snow events on range distribution, we used a chi-square test to determine if the monkeys select the high solar radiation MAPs 1–2 days after snow.

## Supporting Information

Appendix S1 Study site located in southeast Tibetan Plateau, China. The area ranges from 3200 to 4500 m above sea level, characterized by extremely complex topography and climate. The nine winter range MAPs (minimum active polygons) are identified in the northeast corner of the map.
(DOC)

Appendix S2 The pattern of solar radiation from Nov. 1, 2006 to Feb. 10, 2007 (3A, KWH/m^2^) and sunshine duration in Dec. 22, 2006 (3B, min) throughout the monkey's winter range (represented by the 9 MAPs). Areas with red/green color indicate where there is high/low solar radiation and long/short sunshine duration respectively. Note that MAPs #8 and #9 are on opposite sides of a north-south ridge.
(DOC)

## References

[1] Berthold P (2001). Bird Migration: A General Survey..

[2] Canterbury G (2002). Metabolic Adaptation and Climatic Constraints on Winter Bird Distribution.. Ecology.

[3] Boyle WA (2008). Can Variation in Risk of Nest Predation Explain Altitudinal Migration in Tropical Birds?. Oecologia.

[4] Boyle WA, Conway CJ (2007). Why Migrate? A Test of the Evolutionary Precursor Hypothesis.. American Naturalist.

[5] Albon SD, Langvatn R (1992). Plant Phenology and the Benefits of Migration in a Temperate Ungulate.. Oikos.

[6] Igota H, Sakuragi M, Uno H, Kaji K, Kaneko M (2004). Seasonal Migration Patterns of Female Sika Deer in Eastern Hokkaido, Japan.. Ecological Research.

[7] Loiselle BA, Blake JG (1991). Temporal Variation in Birds and Fruits Along an Elevational Gradient in Costa-Rica.. Ecology.

[8] Nicholson MC, Bowyer RT, Kie JG (1997). Habitat Selection and Survival of Mule Deer: Tradeoffs Associated with Migration.. Journal of Mammalogy.

[9] Brinkman TJ, Deperno CS, Jenks JA, Haroldson BS, Osborn RG (2005). Movement of Female White-Tailed Deer: Effects of Climate and Intensive Row-Crop Agriculture.. Journal of Wildlife Management.

[10] Kirkpatrick RC, Long YC, Zhong T, Xiao L (1998). Social Organization and Range Use in the Yunnan Snub-Nosed Monkey *Rhinopithecus Bieti*.. International Journal of Primatology.

[11] Zhao QK, He SJ, Wu BQ, Nash LT (1988). Excrement Distribution and Habitat Use in *Rhinopithecus Bieti* in Winter.. American Journal of Primatology.

[12] Ding W (2003). Feeding Ecology, Social Organization and Conservation Biology of *Rhinopithecus Bieti* at Tacheng, Yunnan [PhD].

[13] Huo S (2005). Diet and Habitat Use of *Rhinopithecus Bieti* at Mt. Longma, Yunnan and Phylogeny of the Family Viverridae in China..

[14] Xiang ZF (2005). The Ecology and Behavior of Black-and-White Snub-Nosed Monkey (*Rhinopithecus Bieti*) at Xiaochangdu in Honglaxueshang National Nature Reserve, Tibet, China..

[15] Hayes JP (1989). Field and Maximal Metabolic Rates of Deer Mice (*Peromyscus Maniculatus*) at Low and High-Altitudes.. Physiological Zoology.

[16] Cui LW (2003). A Note on an Interaction between *Rhinopithecus Bieti* and a Buzzard at Baima Snow Moutain.. Folia Primatologica.

[17] Tibet Forestry Survey and Planning Institute (2000). The Comprehensive Programming to Honglaxueshan National Reserve of Mangkang County..

[18] Cottle HJ (1932). Vegetation on North and South Slopes of Mountains in South-Western Texas.. Ecology.

[19] Holch EE (1931). Development of Roots and Shoots of Certain Deciduous Tree Seedings in Different Forest Sites.. Ecology.

[20] Fu PD, Rich PM (2002). A Geometric Solar Radiation Model with Applications in Agriculture and Forestry.. Computers and Electronics in Agriculture.

[21] Liu ZH, Zhao QK (2004). Sleeping Sites of *Rhinopithecus Bieti* at Mt. Fuhe, Yunnan.. Primates.

[22] Chesser RT, Levey DJ (1998). Austral Migrants and the Evolution of Migration in New World Birds: Diet, Habitat, and Migration Revisited.. American Naturalist.

[23] Chaves-Campos J, Arevalo JE, Araya M (2003). Altitudinal Movements and Conservation of Bare-Necked Umbrellabird *Cephalopterus Glabricollis* of the Tilaran Mountains, Costa Rica.. Bird Conservation International.

[24] Carrascal LM, Diaz JA, Huertas DL, Mozetich I (2001). Behavioral Thermoregulation by Treecreepers: Trade-Off between Saving Energy and Reducing Crypsis.. Ecology.

[25] Huertas DL, Diaz JA (2001). Winter Habitat Selection by a Montane Forest Bird Assemblage: The Effects of Solar Radiation.. Canadian Journal of Zoology-Revue Canadienne De Zoologie.

[26] Wachob DG (1996). The Effect of Thermal Microclimate on Foraging Site Selection by Wintering Mountain Chickadees.. Condor.

[27] Moyer BR, Wagenbach GE (1995). Sunning by Black Noddies (*Anous Minutus*) May Kill Chewing Lice (*Quadraceps Hopkinsi*).. Auk.

[28] Blem CR, Blem LB (1993). Do Swallows Sunbathe to Control Ectoparasites - an Experimental Test.. Condor.

[29] Harris RB, Loggers CO (2004). Status of Tibetan Plateau Mammals in Yeniugou, China.. Wildlife Biology.

[30] Jing Y (2005). Life History, Cooperative Breeding, Foraging Competition and Distribution of Sichuan Jay (*Perisoreus Internigrans*)..

[31] Ma Y, Tian L (2002). Distribution and Population Size of Golden Takin in Niubeiliang Nature Reserve of Shaanxi.. Acta Theriologica Sinica.

[32] Schaller GB, Liu WL (1996). Distribution, Status, and Conservation of Wild Yak *Bos Grunniens*.. Biological Conservation.

[33] Hamilton WJ, Heppner F (1967). Radiant Solar Energy and Function of Black Homeotherm Pigmentation - an Hypothesis.. Science.

[34] Lustick S (1971). Plumage Color and Energetics.. Condor.

[35] IUCN (2010). Iucn Red List of Threatened Species.. http://www.iucnredlist.org/.

[36] Xiao W, Ding W, Cui LW, Zhou RL, Zhao QK (2003). Habitat Degradation of *Rhinopithecus Bieti* in Yunnan, China.. International Journal of Primatology.

[37] Long YC, Kirkpatrick CR, Zhongtai, Xiaolin (1994). Report on the Distribution, Population, and Ecology of the Yunnan Snub-Nosed Monkey (*Rhinopithecus Bieti*).. Primates.

[38] Messerli B, Ives JD (1997). Mountains of the World: A Global Priority..

[39] Liu XD, Cheng ZG, Yan LB, Yin ZY (2009). Elevation Dependency of Recent and Future Minimum Surface Air Temperature Trends in the Tibetan Plateau and Its Surroundings.. Global and Planetary Change.

[40] Cristobal J, Jimenez-Munoz JC, Sobrino JA, Ninyerola M, Pons X (2009). Improvements in Land Surface Temperature Retrieval from the Landsat Series Thermal Band Using Water Vapor and Air Temperature.. Journal of Geophysical Research-Atmospheres.

[41] Panah SKA, Goossens R, Matinfar HR, Mohamadi H, Ghadiri M (2008). The Efficiency of Landsat Tm and Etm+ Thermal Data for Extracting Soil Information in Arid Regions.. Journal of Agricultural Science and Technology.

[42] Zhang J, Wang Y, Wang Z (2007). Change Analysis of Land Surface Temperature Based on Robust Statistics in the Estuarine Area of Pearl River (China) from 1990 to 2000 by Landsat Tm/Etm+ Data.. International Journal of Remote Sensing.

[43] Grueter CC, Li DY, van Schaik CP, Ren BP, Long YC (2008). Ranging of *Rhinopithecus Bieti* in the Samage Forest, China. I. Characteristics of Range Use.. International Journal of Primatology.

[44] Li DY, Grueter CC, Ren BP, Long YC, Li M (2008). Ranging of *Rhinopithecus Bieti* in the Samage Forest, China. Ii. Use of Land Cover Types and Altitudes.. International Journal of Primatology.

[45] Zar JH (1974). Biostatistical Analysis.

