# Supplementary material for: Why Does Rhinopithecus bieti Prefer the Highest Elevation Range in Winter? A Test of the Sunshine Hypothesis
Source: PLoS One. 2011 Sep 7;6(9):e24449. doi: 10.1371/journal.pone.0024449 (PMC3168501; doi:10.1371/journal.pone.0024449)
Supplement: Appendix S1 — Study site located in southeast Tibetan Plateau, China. The area ranges from 3200 to 4500 m above sea level, characterized by extremely complex topography and climate. The nine winter range MAPs (minimum active polygons) are identified in the northeast corner of the map. (DOC) [file pone.0024449.s001.doc]

*
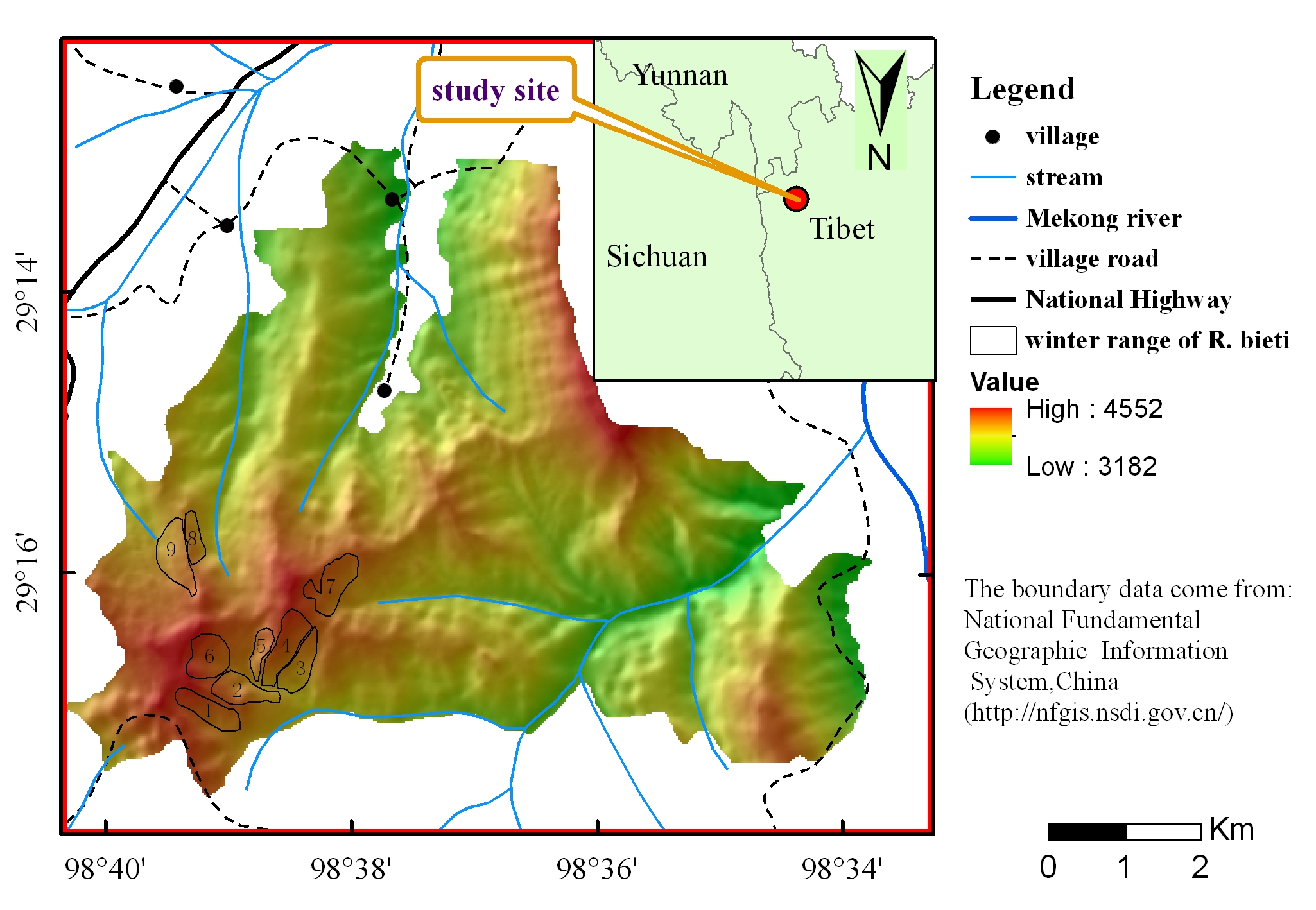
*

Appendix S1: Study site located in southeast Tibetan Plateau, China. The area ranges from 3200 to 4500 m above sea level, characterized by extremely complex topography and climate. The nine winter range MAPs (minimum active polygons) are identified in the northeast corner of the map.
