# Supplementary material for: Why Does Rhinopithecus bieti Prefer the Highest Elevation Range in Winter? A Test of the Sunshine Hypothesis
Source: PLoS One. 2011 Sep 7;6(9):e24449. doi: 10.1371/journal.pone.0024449 (PMC3168501; doi:10.1371/journal.pone.0024449)
Supplement: Appendix S2 — The pattern of solar radiation from Nov. 1, 2006 to Feb. 10, 2007 (3A, KWH/m2) and sunshine duration in Dec. 22, 2006 (3B, min) throughout the monkey's winter range (represented by the 9 MAPs). Areas with red/green color indicate where there is high/low solar radiation and long/short sunshine duration respectively. Note that MAPs #8 and #9 are on opposite sides of a north-south ridge. (DOC) [file pone.0024449.s002.doc]

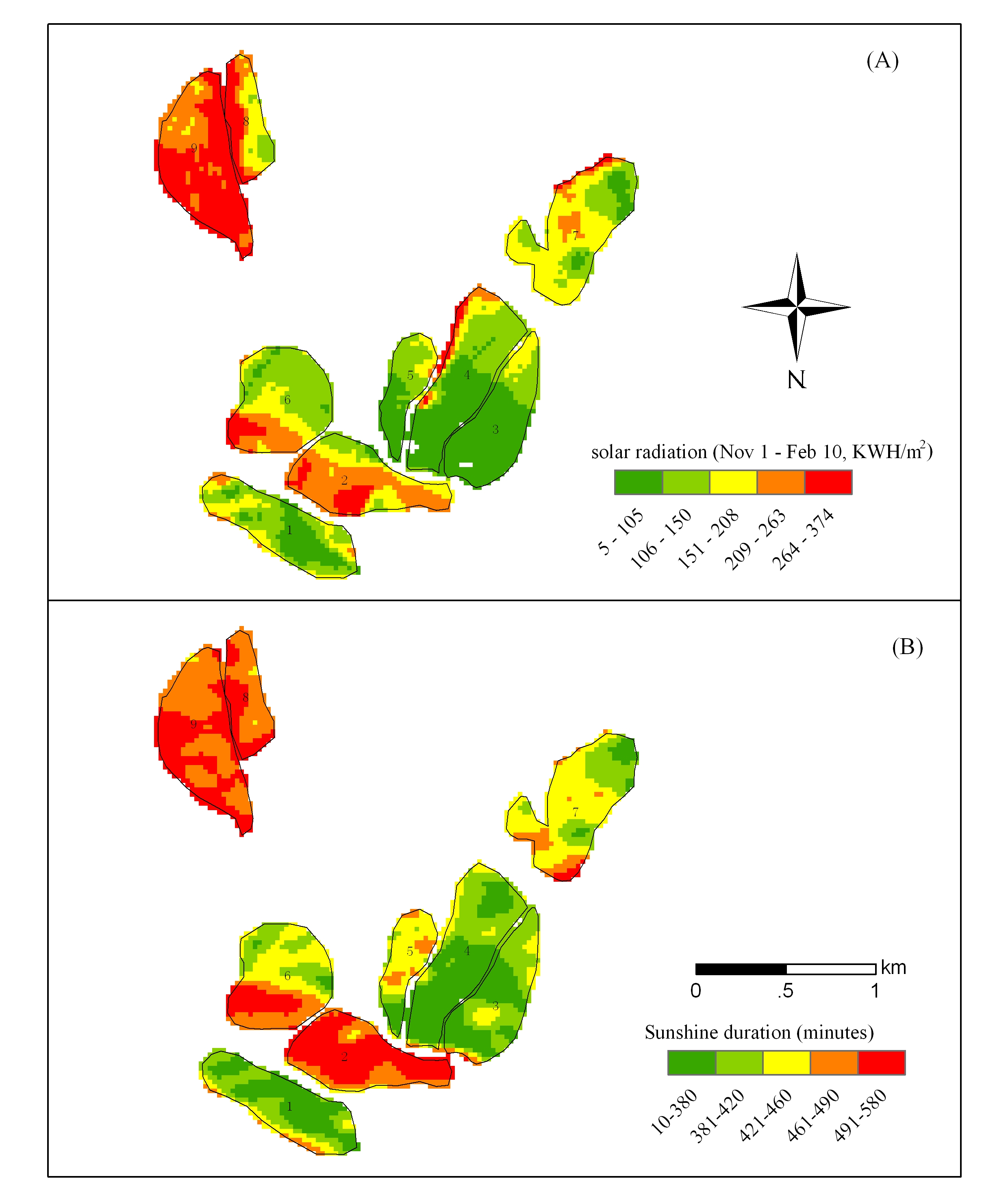


Appendix S2. The pattern of solar radiation from Nov. 1, 2006 to Feb. 10, 2007 (3A, KWH/m^2^) and sunshine duration in Dec. 22, 2006 (3B, min) throughout the monkey’s winter range (represented by the 9 MAPs). Areas with red/green color indicate where there is high/low solar radiation and long/short sunshine duration respectively. Note that MAPs #8 and #9 are on opposite sides of a north-south ridge.
